# Learning Domain-Invariant Representations of Histological Images

**DOI:** 10.3389/fmed.2019.00162

**Published:** 2019-07-16

**Authors:** Maxime W. Lafarge, Josien P. W. Pluim, Koen A. J. Eppenhof, Mitko Veta

**Affiliations:** Medical Image Analysis Group, Department of Biomedical Engineering, Eindhoven University of Technology, Eindhoven, Netherlands

**Keywords:** histopathology image analysis, domain-invariant representation, domain-adversarial convolutional network, mitosis detection, nuclei segmentation

## Abstract

Histological images present high appearance variability due to inconsistent latent parameters related to the preparation and scanning procedure of histological slides, as well as the inherent biological variability of tissues. Machine-learning models are trained with images from a limited set of domains, and are expected to generalize to images from unseen domains. Methodological design choices have to be made in order to yield domain invariance and proper generalization. In digital pathology, standard approaches focus either on *ad-hoc* normalization of the latent parameters based on prior knowledge, such as staining normalization, or aim at anticipating new variations of these parameters via data augmentation. Since every histological image originates from a unique data distribution, we propose to consider every histological slide of the training data as a domain and investigated the alternative approach of domain-adversarial training to learn features that are invariant to this available domain information. We carried out a comparative analysis with staining normalization and data augmentation on two different tasks: generalization to images acquired in unseen pathology labs for mitosis detection and generalization to unseen organs for nuclei segmentation. We report that the utility of each method depends on the type of task and type of data variability present at training and test time. The proposed framework for domain-adversarial training is able to improve generalization performances on top of conventional methods.

## 1. Introduction

The traditional microscopy-based workflow of pathology labs is undergoing a rapid transformation since the introduction of whole-slide scanning. This new technology allows viewing of digitized histological slides on computer monitors and integration of advanced image analysis algorithms, which can enable pathologists to perform more accurate and objective analysis of tissue.

The process of producing a digital slide consists of several successive procedures: formalin fixation and paraffin embedding of the tissue, sectioning, staining and scanning. Each procedure has a multitude of parameters that vary between pathology labs and within the same lab over time. This results in significant tissue appearance variation in the digital slides, that adds to the underlying biological variability that can occur, for example, due to differences in tissue type or pathology.

In a real-world scenario, histological images are made available in pair with ground-truth annotations for the development of a predictive model to solve and automate a given task. Very often, these images were acquired in specific conditions (via the same scanner, following a lab-specific preparation process or from a small cohort for example) resulting in a narrower range of appearances than what could be observed in other conditions (different scanner, lab or cohort).

The discrepancy between the restricted data distribution available at training time and the higher variability of possible histological images on which a model is expected to perform, often limits the generalization of image analysis techniques, including deep learning-based methods.

This problem is typically addressed with *ad-hoc* methods based on known priors. For instance, one might correct for the known staining variability via a staining normalization approach. However, relying on such specifically chosen priors raises the risk to leave out or enhance domain-specific noise in the learned representation. For example, staining normalization methods will not handle other sources of variability such as specific tissue pleomorphism.

Deep learning methods learn abstract representations directly from the image data and have achieved state-of-the-art results in many computer vision and medical image analysis tasks including histopathology. Every histological slide results from a given set of latent parameters (corresponding to a specific case, hospital or tissue type for instance) and thus can be considered an individual domain. As such, all the image patches extracted from a given whole slide image (WSI) are samples of the same data distribution, and so, the same domain. We hypothesize that learning a representation that is explicitly invariant to the domains of the training data is likely to be also invariant, to some extent, to new unseen domains.

**Figure 1:**
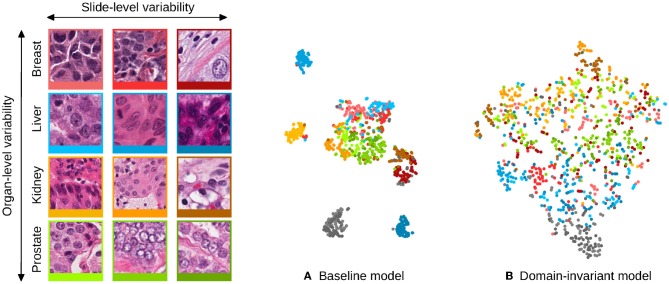
Illustration of the domain distribution of the internal representation of convolutional neural networks (CNN) trained for the task of nuclei segmentation. The scatter plots are t-SNE embeddings (1) of a random selection of 64 image patches for each of 12 digital slides, for which a representative patch is displayed on the left and framed with matching colors. Each image patch is represented by the concatenated means and standard deviations of its activations after the second convolutional layer of the CNNs. Two models are compared: **(A)** shows the representation learned by a baseline CNN model and **(B)** a model that uses stain-normalized inputs and domain-adversarial training. The models were trained with image patches from these 12 slides, and image patches from two hold-out slides (colon tissue type) were embedded the same way and are shown in gray. The baseline model induces domain clusters in the embedding space whereas the domain-invariant model produces a smoother distribution of the domains.

This hypothesis is motivated by the fact that regular Convolutional Neural Networks (CNNs) preserve domain information in their representation that is not useful for the task at hand. This phenomenon is illustrated in Figure 1A: the appearance features present in some digital slides form separated clusters in the space of the learned representation, even if the slides share a known variability factor (patches from different liver tissue images, in blue, are distributed apart when represented by a baseline model). In the example of Figure 1A, image patches that originate from an unseen domain (colon tissue represented in gray), form a disjoint cluster, in a region that the model was not trained to process, and that is likely to lead to poor performances. However, this internal distribution can become smoother when strategies are employed to make the representation domain-invariant. The distribution of the embeddings shown in Figure 1B, illustrates how the representation of seen domains that was disjoint among the same organ now overlaps, and how unseen domains align with this smooth distribution: the gray cluster representing an unseen organ tissue type is now connected to the rest of the embeddings, and is more likely to lead to better generalization performances.

In this paper, we propose a domain-adversarial framework to constrain CNN models to learn domain-invariant representations (section 3.2.2), and compare it with staining normalization (section 3.3.2), augmentation methods (sections 3.3.1 and 3.3.3) and combinations of these methods.

Domain-adversarial training differs from conventional methods in the sense it does not rely on defined hard priors: the proposed framework leverages the domain information that is available in most histopathology datasets in order to achieve domain invariance, whereas this information is usually left aside by conventional methods.

This work is an extension of the comparative analysis presented at the 2017 MICCAI-DLMIA workshop (2). In addition to an extended set of experiments, we also make a novel technical contribution that enables the use of batch normalization when training a single network with different input data distributions, as is required with domain adversarial networks.

We show experiments for two different tasks: (1) mitosis detection with a testing set originating from pathology labs that were unseen during training and (2) nuclei segmentation with a testing set consisting of tissue types that were unseen during training.

## 2. Related Work

Machine learning models for histopathology image analysis that directly tackle the appearance variability can be grouped in two main categories: (1) methods that rely on pre-processing of the image data and (2) methods that directly modify the machine learning model and/or training procedure.

The first group of methods includes a variety of staining normalization techniques (3, 4). Some image processing pipelines handle the variability problem via extensive data augmentation strategies, often involving color transformations (2, 5–8). Hybrid strategies that perturb the staining distributions on top of a staining normalization procedure have also been investigated (9–12).

The second group of methods is dominated by domain adaptation approaches. Domain adaptation assumes the model representation learned from a source domain can be adapted to a new target domain. Fine-tuning and domain-transfer solutions were proposed for deep learning models (13–16), and with applications to digital pathology (17–19). Another approach consists in considering the convolutional filters of the CNN as domain-invariant parameters whereas the domain variability can be captured with the Batch Normalization (BN) parameters (20, 21). Adaptation to new domains can be achieved by fine-tuning a new set of BN parameters dedicated to these new domains (21).

Adversarial training of CNNs was proposed to achieve domain adaptation from a source domain of annotated data to a single target domain from which unlabeled data is available (22). Adversarial approaches aim at learning a shared representation that is invariant to the source and target domains via a discriminator CNN, that is used to penalize the model from learning domain-specific features (22–26). This type of method has been successfully applied and adapted to the field of medical image analysis (27). These methods, however, require that data from the target domains is available at training time, which is not a constraint of our approach and were not investigated on tasks involving histological images. Finally, we proposed in Lafarge et al. (2) a similar approach that enforces the model to learn a domain-agnostic representation for a given extensive domain variability present within the training data and we investigated its ability to perform on new unseen domains.

## 3. Materials and Methods

We evaluate the different approaches for achieving domain invariance on two relevant histopathology image analysis tasks: nuclei segmentation and mitosis detection. Automated nuclei segmentation is an important tool for many downstream analyses of histopathological images, such as assessment of nuclear pleomorphism. Mitosis detection is the first step toward assessment of the tumor proliferation activity, and is therefore an important biomarker for breast cancer prognostication and part of the widely used Bloom-Richardson-Elston grading system (28).

In this section, we first describe the datasets used for the two image analysis tasks, and specify the domain shift under which the generalization of trained models on new domains will be assessed. Then, we describe the baseline convolutional neural network model, the domain-adversarial framework, the staining normalization and the data augmentation approaches that will be used in the comparative analysis.

### 3.1. Datasets

The proposed comparative analysis was made on two datasets which expose two different types of domain variability. These datasets correspond to different tasks, enabling to study the framework viability in multiple analysis settings.

#### 3.1.1. Inter-lab Mitosis Dataset

We used the TUPAC16 dataset (29) that includes 73 breast cancer cases with histological slides stained with Hematoxylin-Eosin (H&E). The dataset consists of a selection of high power field images (HPF) that were annotated with mitotic figure locations, derived from the consensus of at least two pathologists.

The cases come from three different pathology labs (PL_A_, PL_B_ and PL_C_ with 23, 25, and 25 cases, respectively) and were scanned with two different whole-slide image scanners (the slides from PL_B_ and PL_C_ were scanned with the same scanner). We split the dataset as follows:

- A training set of eight cases from PL_A_ (458 mitoses).
- A validation set with four other cases from PL_A_ (92 mitoses).
- A test set with the remaining 11 cases from PL_A_ (533 mitoses), in order to measure the intra-lab performance of the trained models in the same condition as the AMIDA13 challenge (30).
- A test set using the 50 cases from PL_B_ and PL_C_ (469 mitoses), in order to evaluate inter-lab generalization performance.

#### 3.1.2. Multi-Organ Nuclei Dataset

We used the multi-organ dataset created in (4): a subset of 30 HPF images, selected from single WSIs of H&E-stained tissue slices, prepared in 18 different hospitals, and provided by The Cancer Genome Atlas (31). These 30 images consist of seven different tissue types with nuclei mask annotations publicly available (4).

To be in conditions similar to (4), we split the dataset in two groups of tissue types T_A_={*Breast, Liver, Kidney, Prostate*} and T_B_={*Bladder, Colon, Stomach*}. For experimental purpose, we split the dataset in the conditions of (4) as follows:

- A training set of 12 HPF images with three images for each tissue type of T_A_ (7337 nuclei).
- A validation set of 4 other HPF images with one images for each tissue type of T_A_ (1474 nuclei).
- A test set of 8 other HPF images with 2 images for each tissue type of T_A_ (4130 nuclei).
- A test set using the 6 HPF images of T_B_ with 2 images of each type (4025 nuclei), in order to evaluate cross-tissue-type generalization performance.

### 3.2. Domain-Adversarial Framework

The framework we propose is designed for classification tasks given images **x** that are associated with class labels **y**.

#### 3.2.1. The Underlying Convolutional Network

**Figure 2:**
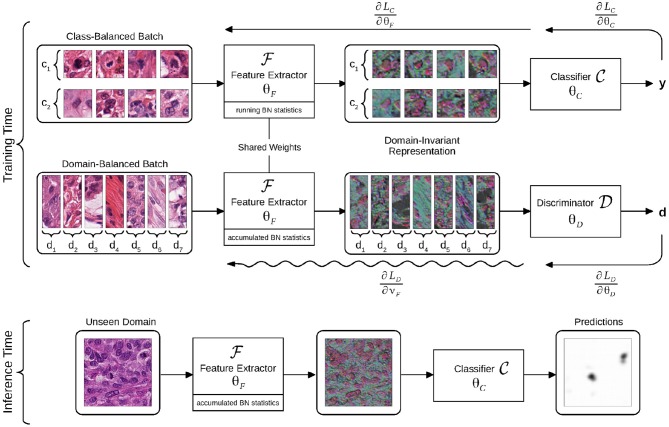
Flowchart of the domain-adversarial model. The model is trained using batches balanced across classes or domains. The intermediate representation is learned to optimize the classifier, while the discriminator is trained to identify domains of origin from this representation. Gradient back-propagations are shown with right-to-left arrows, adversarial back-propagation is shown with a sinuous arrow. The domain-invariant representation is illustrated as a selection of three activation maps that are output by the feature extractor, all assigned to RGB channels.

The proposed framework is applicable to any baseline CNN architecture that can be decomposed in two parts: a feature extractor CNN F and a classifier CNN C, parameterized by ***θ***_*F*_ and ****θ****_*C*_, respectively, as illustrated in Figure 2.

F takes images **x** as input and outputs an intermediate representation $F\left(\text{x};\theta_{F}\right)$, whereas C takes $F\left(\text{x};\theta_{F}\right)$ as input and outputs a classification probability $C\left(F\left(\text{x};\theta_{F}\right);\theta_{C}\right)$. The (F, C) pipeline can be trained by minimizing the cross-entropy loss $L_{C}\left(\text{x},\text{y};\theta_{F},\theta_{C}\right)$. ***θ***_*F*_ and ***θ***_*C*_ are optimized by stochastic gradient descent using mini-batches of image-label pairs (**x**, **y**).

#### 3.2.2. Domain-Adversarial Training

The goal of the framework is to make the intermediate representation $F\left(\text{x},\theta_{F}\right)$ invariant to the domains of the training data. We make the assumption that by making $F\left(\text{x},\theta_{F}\right)$ domain-agnostic, this will improve the cross-domain generalization of the classifier C. By making the representation invariant to the known domain variability of the training data, we can expect, in some extent, it will also be invariant to unseen variability factors.

Toward this goal, we turned the baseline CNN to a domain-adversarial neural network (DANN) (22) by involving a discriminator CNN D with parameters ***θ***_*D*_. D takes the representation $F\left(\text{x};\theta_{F}\right)$ as input and predicts the domain probability $D\left(F\left(\text{x};\theta_{F}\right),\theta_{D}\right)$ of the input training images **x** via softmax activation. We define $L_{D}\left(\text{x},d;\theta_{F},\theta_{D}\right)$ as the cross-entropy loss of the domain discriminator given an input of image-domain pair (**x**, *d*), with *d* a domain identifier, unique to each slide of the training dataset.

The minimization of $L_{D}\left(\text{x},d;\theta_{F},\theta_{D}\right)$ during training implies that domain-specific features get extracted from the shared representation that we want to make domain-invariant. Such domain identification is possible since regular models naturally distribute domains apart in the representation as shown in Figure 1A. In order to obtain domain-invariance, the weights ***θ***_*F*_ are jointly optimized by stochastic gradient ascent, to maximize $L_{D}\left(\text{x},d;\theta_{F},\theta_{D}\right)$. This process aims at removing domain-specific features from the representation that are useless for the task at hand, as it is illustrated in Figure 1B, while still being optimized to improve the performances of C.

#### 3.2.3. Handling Classification-Related and Domain-Related Input Distributions

Batch Normalization (BN) (32) is used throughout the networks F, C, and D as it is an efficient method that allows fast and stable training, in particular with adversarial components (33). By normalizing every batch with computed mean and variance at every convolutional layer, BN implies that the distribution of the feature maps is a function of the distribution of the input batch. As a consequence, the distribution of the feature maps will vary with the balance of the batch associated with every pass (see section 3.2.2).

It is necessary for the domain-adversarial update to be computed with a forward-pass in the same conditions as for the classification pass. Therefore, we propose to apply BN during the adversarial pass using the accumulated moments of F, while keeping a regular BN computation and regular moment accumulation during the classification pass. To this end, we adjusted the adversarial update (4) to update only the convolutional weights **ϑ**_*F*_ ⊂ ***θ***_*F*_, so as not to interfere with the BN weights, updated according to (1) of the classification pass, with a similar motivation as in Karani et al. (21).

The domain-adversarial training procedure consists in alternating between four update rules:

Optimization of the feature extractorwith learning rate λ_C_:

(1) $$\begin{array}{r} \theta_{F}\leftarrow \theta_{F}-\lambda_{C}\frac{\partial L_{C}}{\partial \theta_{F}} \end{array}$$

Optimization of the classifier:

(2) $$\begin{array}{r} \theta_{C}\leftarrow \theta_{C}-\lambda_{C}\frac{\partial L_{C}}{\partial \theta_{C}} \end{array}$$

Optimization of the domain discriminatorwith learning rate λ_D_:

(3) $$\begin{array}{r} \theta_{D}\leftarrow \theta_{D}-\lambda_{D}\frac{\partial L_{D}}{\partial \theta_{D}} \end{array}$$

Adversarial update of the feature extractor:

(4) $$\begin{array}{r} \vartheta_{F}\leftarrow \vartheta_{F}+\alpha \lambda_{D}\frac{\partial L_{D}}{\partial \vartheta_{F}} \end{array}$$

The update rules (1) and (4) work in an adversarial way: with (1), the parameters ***θ***_*F*_ are updated for the classification task (by minimizing $L_{C}$), and with (4), a subset of the same parameters are updated to prevent the domains of origin to be recovered from the representation $F\left(\cdot ;\theta_{F}\right)$ (by maximizing $L_{D}$). The parameter α ∈ [0, 1] controls the influence of the adversarial component.

### 3.3. Comparison of Methods

**Figure 3:**
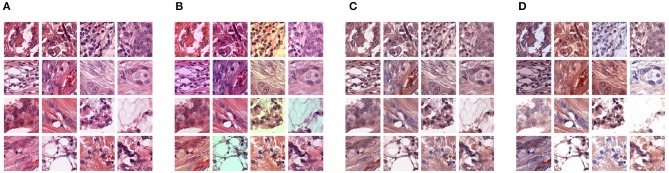
Illustration of different types of pre-processing augmentations: **(A)** original images, **(B)** RGB color augmentation, **(C)** staining normalization, **(D)** staining augmentation.

For comparison purpose, we chose to study three different well-established standard methods that aim at improving the generalization of deep learning models in the context of histopathology image analysis and that do not require additional data. A visual overview of these methods is presented in Figure 3. We also analyzed combinations of these individual approaches together with the proposed domain-adversarial training framework.

#### 3.3.1. Color Augmentation

Since the most prominent source of variability in histology images is the staining color appearance, one alternative to artificially produce new training samples consists in randomly perturbing the color distribution of sampled image patches. By increasing the amount of different color distributions in the training set, the model is expected to learn a representation that better generalize to this type of variability.

We performed color augmentation (CA) by transforming the contrast and shifting the intensities of every color channel *I*_*c*_ ← *a*_*c*_ · (*I*_*c*_ − μ(*I*_*c*_)) + μ(*I*_*c*_) + *b*_*c*_, where *a*_*c*_ and *b*_*c*_ are drawn from uniform distributions *a*_*c*_ ~ *U* [0.9, 1.1] and *b*_*c*_ ~ *U* [−13, +13] and where μ(*I*_*c*_) is the mean intensity of *I*_*c*_.

#### 3.3.2. Staining Normalization

The opposite strategy is to reduce the appearance variability of all the images as a pre-processing step before training and evaluating a trained CNN model. For hematoxylin and eosin (H&E) stained slides, staining normalization (SN) methods can be used (34, 35).

The RBG pixel intensities of H&E-stained histopathology images can be modeled with the Beer-Lambert law of light absorption: *I*_*c*_ = *I*_0_ exp(−**A**_*c*,∗_ · **C**). In this expression *c* = 1, 2, 3 is the color-channel index, **A** ∈ [0, +∞]^3×2^ is the matrix of absorbance coefficients and **C** ∈ [0, +∞]^2^ are the stain concentrations (34). We perform staining normalization with the method described in (35). This is an unsupervised method that decomposes any image with estimates of its underlying **A** and **C**. The appearance variability over the dataset can then be reduced by recomposing all the images using some fixed reference absorbance coefficients **A**_*ref*_.

#### 3.3.3. Staining Augmentation

An approach between CA and SN consists in artificially perturbing the distribution of the concentrations estimated in the unmixing step of SN before applying the recomposing step with constant **A**_*ref*_ (9–12).

We experimented with Staining Augmentation (SNA) for comparison, by randomly perturbing each estimated concentration map **C**_*i*_ linearly with **C**_*i*_ ← *g*_*i*_ · **C**_*i*_ + *h*_*i*_, where *g*_*i*_ and *h*_*i*_ are drawn from uniform distributions *g*_*i*_ ~ *U* [0.9, 1.1] and *h*_*i*_ ~ *U* [−0.1, +0.1].

## 4. Experiments

We implemented two DANN models (22), one for the mitosis detection task and one for the nuclei segmentation task. Both problems are approached with a patch-based classification setup. In the case of mitosis detection, C outputs the probability for the input patches to be centered on mitotic figures. In the case of nuclei segmentation, C outputs the 3-class probability vectors for the center of the image patches: nuclei foreground, nuclei edge, or background.

### 4.1. Architectures

For both problems, we chose straightforward convolutional networks, similar to the related literature (2, 4, 29, 30, 36). We chose to investigate DANN models with a single bifurcation at the second max-pooling layer, corresponding to receptive fields of size 12 × 12 for the mitosis classifier and 16 × 16 for the nuclei classifier.

Every convolutional layer is activated by a leaky Rectified Linear Unit (with coefficient 0.01), except for the output layers that are activated by a softmax function. Architecture details are presented in Tables 1, 2.

**Table 1:** Architecture of the mitosis detection model.

**Feature extractor and mitosis classifier**					**Domain classifier**				
	**Layer**	**Size**	**Filter**	**Rec. F**.	**Layer**	**Output**	**Filter**	**Rec. F**.	
F	**Input**	64 × 64 × 3		1 × 1					
	Conv	60 × 60 × 16	5 × 5	5 × 5					
	Max pool	30 × 30 × 16	2 × 2	6 × 6					
	Conv	28 × 28 × 16	3 × 3	10 × 10					
	Max pool	14 × 14 × 16	2 × 2	12 × 12	**Bifurcation**	14 × 14 × 16		12 × 12	
C	Conv	12 × 12 × 16	3 × 3	20 × 20	Conv	12 × 12 × 32	3 × 3	20 × 20	D
	Max pool	6 × 6 × 16	2 × 2	24 × 24	Conv	10 × 10 × 64	3 × 3	24 × 24	
	Conv	4 × 4 × 16	3 × 3	40 × 40	**Softmax**	10 × 10 × 8	1 × 1	24 × 24	
	Max pool	2 × 2 × 16	2 × 2	48 × 48					
	Conv	1 × 1 × 64	2 × 2	64 × 64					
	**Sigmoid**	1 × 1 × 1	1 × 1	64 × 64					

*The feature extractor F and mitosis classifier C form a 10-layer CNN with a single class-probability output. The domain classifier D is a 3-layer network bifurcated at the second max-pooling layer of F and outputs a 8-domain probability vector*.

**Table 2:** Architecture of the nuclei segmentation model.

**Feature extractor and nuclei classifier**					**Domain classifier**				
	**Layer**	**Size**	**Filter**	**Rec. F**.	**Layer**	**Output**	**Filter**	**Rec. F**.	
F	**Input**	52 × 52 × 3		1 × 1					
	Conv	48 × 48 × 24	5 × 5	5 × 5					
	Max pool	24 × 24 × 24	2 × 2	6 × 6					
	Conv	20 × 20 × 24	5 × 5	14 × 14					
	Max pool	10 × 10 × 24	2 × 2	16 × 16	**Bifurcation**	10 × 10 × 24		16 × 16	
C	Conv	8 × 8 × 24	3 × 3	24 × 24	Conv	8 × 8 × 32	3 × 3	24 × 24	D
	Max pool	4 × 4 × 24	2 × 2	28 × 28	Conv	6 × 6 × 64	3 × 3	28 × 28	
	Conv	2 × 2 × 24	3 × 3	44 × 44	**Softmax**	6 × 6 × 12	1 × 1	28 × 28	
	Conv	1 × 1 × 96	2 × 2	52 × 52					
	**Softmax**	1 × 1 × 3	1 × 1	52 × 52					

*The feature extractor F and nuclei classifier C form a 9-layer CNN with a 3-class probability output. The domain classifier D is a 3-layer network bifurcated at the second max-pooling layer of F and outputs a 12-domain probability vector*.

### 4.2. Training Procedures

We used the same training procedure for the models of both the problems. For all experimental configurations, image patches were transformed by a baseline augmentation pipeline consisting of a random 90-degree rotation, random mirroring, −10/ + 10% spatial-scaling. Sampling of non-mitosis figures and nuclei background classes were adjusted by hard-negative mining using a first version of the baseline models to reject easy-to-classify image patches. The domain-balanced batches were built using patches of size 24 × 24 for the mitosis detection model and 28 × 28 for the nuclei segmentation model in order for the domain classifiers to output 1 × 1 predictions.

The model weights were optimized with Stochastic Gradient Descent with learning rates λ_*C*_ = 0.01 and λ_*D*_ = 0.001 and momentum μ = 0.9. λ_*C*_ and λ_*D*_ were decayed by a factor of 0.9 every 5,000 iterations. *L*_2_-regularization was applied to all the convolutional weights. For stability purposes and as proposed in (27), we used a warm-up scheduling for the coefficient α, to control the influence of the adversarial component, by following a linear increase from 0.0 to 1.0 from the 5000th to the 10000th training iteration.

## 5. Results

This section presents quantitative and qualitative evaluations of the ability of the developed models to generalize to a known factor of variability of the test set that is absent from the training data.

### 5.1. Mitosis Detection

The performances of the mitosis detection models were evaluated with the F1-score as described in Veta et al. (29, 30), and Cireşan et al. (36). We used the trained classifiers to produce dense mitosis probability maps for all test images. All local maxima above an operating point were considered detected mitotic figures. This operating point was determined as the threshold that maximizes the F1-score over the validation set.

**Figure 4:**
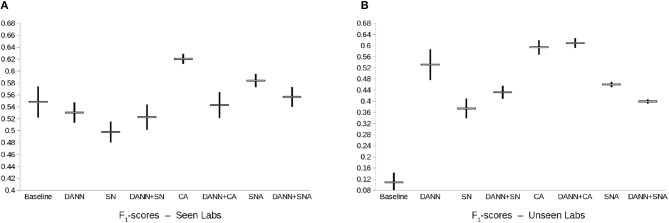
Box-plot of the F1-score of the mitosis classification models. Points represent the mean ± standard deviation of the F1-score of each model across 3 repeats with random initialization and random patch sampling. DANN, Domain-Adversarial Neural Network; CA, Color Augmentation; SN, Staining Normalization; SNA, Staining Augmentation.

On the test made of images acquired in the same labs as the images of the training set, all methods and combinations have relatively good performances, in line with previously reported results (5, 29, 30, 36). The best performing method is CA (F1-score of 0.62 ± 0.008, see Figure 4). Adding domain-adversarial training does not improve performance of the conventional methods.

On the other hand, on the test set of images acquired in different labs than for the training set, the best performing method is the combination of CA and DANN (F1-score of 0.609 ± 0.017). The baseline model does not generalize properly to unseen labs, and domain-adversarial training improves the performances except for the combination with SNA.

### 5.2. Nuclei Segmentation

We used the trained nuclei classifiers to produce segmented nuclei objects. First we generated a set of object seeds using the object foreground map prediction, thresholded by an operating point selected based on a validation set. A set of background seeds were generated using the background prediction, thresholded by a constant of 0.5. Finally a set of segmented nuclei objects was generated using the watershed algorithm given the computed background and foreground seeds and the predicted edges as the topographic relief.

All segmented objects with more than 50% overlap with ground-truth annotations were considered as hits. The performances of the nuclei segmentation models were evaluated with the F1-score as described in (4), computed over a whole test set.

**Figure 5:**
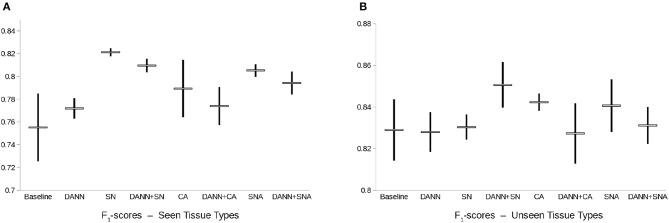
Box-plot of the F1-score of the nuclei segmentation models. Points represent the mean ± standard deviation of the F1-score of each model across 3 repeats with random initialization and random patch sampling. DANN, Domain-Adversarial Neural Network; CA, Color Augmentation; SN, Staining Normalization; SNA, Staining Augmentation.

On the test set of images of seen tissue types, the best performing method is SN (F1-score of 0.821 ± 0.004, see Figure 5). On the test set of unseen tissue types, the best performing method is the combination of SN and domain-adversarial training (F1-score of 0.851 ± 0.011). On both test sets, domain-adversarial training produces a decrease in generalization performance when combined with augmentation methods (CA and SNA).

The baseline model generalizes properly due to the high variability already present in the training set, and therefore is in line with the results reported in Kumar et al. (4). We report a difference of the range of performances between the two test sets.

### 5.3. Qualitative Results

**Figure 6:**
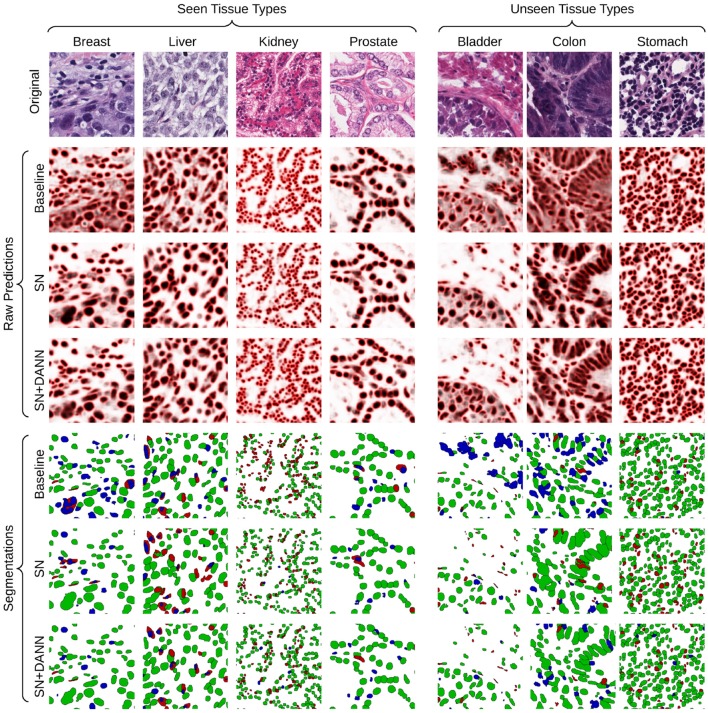
Visualization of the raw predictions (background: white, foreground: black, border: red) and resulting segmentation maps of the baseline and best-performing segmentation models. True positive, false positive and false negative are show in green, blue, and red, respectively.

Qualitatively, we observe that the baseline models fail to generalize with images that have unseen low-contrast appearance (see *Bladder* and *Colon* examples in Figure 6). This limit is solved by methods involving staining normalization. The addition of domain-adversarial training tends to better separate touching nuclei, improving the F1-score.

**Figure 7:**
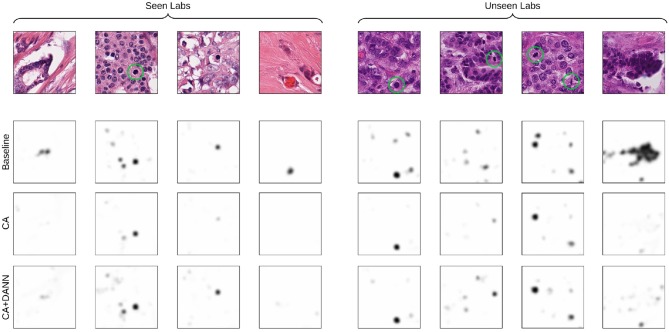
Visualization of the predictions of the baseline and best-performing models (CA for seen-lab test set, and CA+DANN for unseen-lab test set). Ground-truth mitotic figures are circled in green.

Likewise, low-contrast structures occurring in the images from the unseen labs entail false positive detection of mitotic figures (see Figure 7), whereas these do not occur for models trained using CA. The addition of domain-adversarial training tends to produce smoother distribution of the predictions, resulting in a higher rate of true positives and higher F1-score.

## 6. Discussion and Conclusions

The relative improvement of performances brought by the analyzed methods depends on the data and task at hand. In the case where the training data presents a high domain variability (images from multiple labs, multiple organ types), SN is the most effective method when testing on the test set that consists of the same tissue types, because the model can learn to be efficient to the range of staining variability observed on these specific tissue types. However, CA and SNA generalize better than SN on the test set that consists of unseen tissue types as they allow to anticipate the new color and staining variability that can occur in these images. We assume this limit of SN is overcome when combined with domain-adversarial training, as it enables the model to improve the generalization of the learned representation beyond the range of staining distributions seen in the training set.

In the case where the training data presents a low domain variability (intra-lab variability only), CA and SNA, were the most effective methods when testing on unseen images whether they were obtained in the same lab as for the training data or in different labs. This implies that augmentation methods or domain-adversarial training can better anticipate unseen color/staining distributions than SN in this situation. The failure case of the baseline model indicates *overfitting* to the limited variability of domains of the training data and is avoided by CA, SNA or domain-adversarial training. The additional improvement of performances shown when domain-adversarial training is combined with CA indicates that this approach helps the model to generalize to factors other than colors.

Two design choices need to be considered in the proposed domain-adversarial framework as we assume they have an influence on the task performances. These parameters depend on the type of image, task at hand and type of domain variability, and thus need to be carefully tuned.

First, the depth level of F has to be chosen: with an early bifurcation, the low-level features can be made invariant (with the risk of over-fitting to the domains of the training data), whereas a late bifurcation can make the high-level features invariant with the risk that the early features do not get affected by the domain-adversarial update, thus failing to extract features in unseen domains. Fine-tuning this hyper-parameter is necessary to obtain optimal performances. An alternative solution could consist in using multiple bifurcations as it was proposed in Lafarge et al. (2) and Kamnitsas et al. (27).

The receptive field of D on the input is another point to consider. Depending on the task at hand, the receptive field of D does not need to be necessarily the same as C, especially if the source of domain variability can be captured in a field of view smaller than the objects that are being classified. Using too large a receptive field for D raises the risk of identifying, and removing from the representation, some features specific to a domain that might actually be relevant for the task at hand.

In conclusion, we proposed a domain-adversarial framework for training CNN models on histopathology images, and we made a comparative analysis against conventional pre-processing methods. We showed that exploiting slide-level domain information at training time, via an adversarial training process, is thus a suitable additional approach toward domain-invariant representation learning and to improve generalization performances. Still, the performances of a trained model vary with the type of normalization/augmentation method used and the type of variability present in the data at training and inference time. Analyzing these factors is therefore a critical decision step when designing machine-learning models for histology image analysis. Directions for further research include adapting the framework to other model architectures, other tasks, and exploiting known variability factors other than slide-level information. The relative top-performances that domain-adversarial training achieved, confirm it is a relevant research direction toward a general method for consistent generalization to any type of unseen variability of histological images.

## Data Availability

The datasets used for this study are publicly available. For more details, see Veta et al. (29) for the inter-lab mitosis dataset, and Kumar et al. (4) for the multi-organ nuclei dataset.

## Author Contributions

ML and MV contributed to the conception and design of the study. ML implemented the experiments and wrote the first draft of the manuscript. All authors contributed to the result analysis, manuscript revision, read and approved the submitted version.

### Conflict of Interest Statement

The authors declare that the research was conducted in the absence of any commercial or financial relationships that could be construed as a potential conflict of interest.
